# Electroacupuncture as a tool to stimulate bone marrow megakaryocytes in mice: A pilot study

**DOI:** 10.1016/j.htct.2025.103966

**Published:** 2025-08-23

**Authors:** Luiza P.R. dos Santos Mariani, Rita M.V.M. Rocha, Lidiane M.B. Leite, Alexandra C. Senegaglia, Pedro V. Michelotto

**Affiliations:** a Graduate Program in Animal Science, Pontifícia Universidade Católica Paraná, Rua Imaculada Conceição 1155, Prado Velho, 80215-901, Curitiba, PR, Brazil; b Veterinary Medicine, Pontifícia Universidade Católica do Paraná, Curitiba, PR, Brazil; c Core for Cell Technology, Pontifícia Universidade Católica do Paraná, Curitiba, PR, Brazil

Dear Editor,

We report the first evidence that electroacupuncture stimulates megakaryocyte production in the bone marrow (BM) of mice, offering a novel approach to support megakaryopoiesis and improve animal research.

Platelets, derived from megakaryocytes, are critical not only for hemostasis but also for vascular integrity, inflammation, pathogen defense, and tissue repair. They orchestrate innate and adaptive immune responses by expressing Toll-like receptors, activating leukocytes, releasing defensins, and engaging the complement system [1,2]. Enhancing platelet production is particularly important in thrombocytopenia and BM suppression [2,3].

In mouse models, BM sampling typically involves euthanasia and femur dissection, limiting longitudinal evaluations and conflicting with the 3Rs principles (replacement, reduction and refinement) [4, 5, 6]. We aimed to test whether electroacupuncture, a modern adaptation of Traditional Chinese Medicine, stimulates megakaryocyte production and to validate a minimally invasive iliac crest aspiration technique that permits repeated sampling in the same animal. We studied 30 six-month-old male BALB/c mice (15–22 g), housed individually under controlled conditions (20 °C, 12/12-h light-dark cycle, 70 % humidity, ad libitum food and water). Mice were randomized into three groups (*n* = 10 each): control group, electroacupuncture-treated (Electroacupuncture group), and sham electroacupuncture-treated (Sham group). Electroacupuncture was applied at large intestine (LI)-4 and LI-11, bladder (BL)-12 and BL-13, governing vessel (GV)-14 and GV-20 acupoints using sterile stainless-steel needles (0.16 mm × 9 mm, 0.18 mm × 8 mm; DUX®, Brazil). Treatments were performed under general anesthesia, using alternating currents (2 Hz/50 Hz) with 10s/30 s stimulation cycles and 5 s breaks at 2.0 mA, for 45 min, repeated over two weeks. Animals of the Sham group received identical stimulation at non-meridian points. Treatments were conducted by a veterinary acupuncture specialist (Figure 1). BM aspiration was performed under anesthesia on Days 0 and 44 by exposing the iliac crest through a 0.5 cm skin incision and aspirating up to 0.5 % of body weight. Postoperative care included monitoring and analgesia. BM smears were stained with May-Grünwald-Giemsa, and megakaryocyte counts were analyzed histologically. Statistical comparisons were conducted using the Mann-Whitney U and Wilcoxon tests (GraphPad Prism 9.4.1; p-value <0.05). Ethics approval was granted by the Pontifícia Universidade Católica do Paraná Animal Ethics Committee (registration 1247).

**Figure 1:**
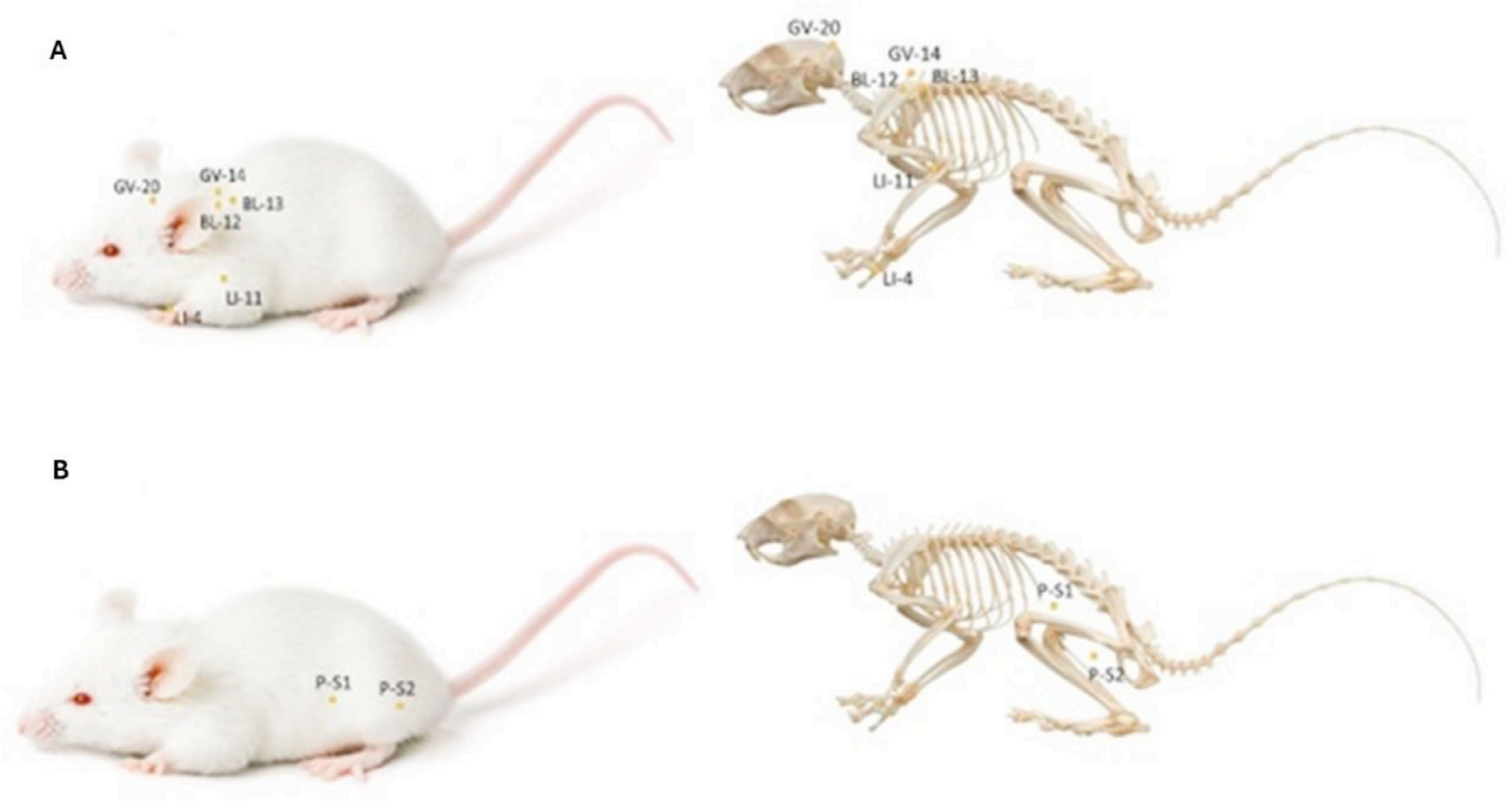
Locations of electroacupuncture and sham electroacupuncture points in BALB/c mice. A) Electroacupuncture points: LI-4 (between the first and second metacarpal bones), LI-11 (lateral to the elbow crease), BL-12 and BL-13 (paraspinal region, at thoracic vertebral levels), GV-14 (between the spinous processes of C7 and T1), and GV-20 (at the midline of the skull). B) Sham electroacupuncture points: P-S1 (lateral abdomen, on the transverse abdominal muscle at the level of the 11th rib) and P-S2 (lateral hindlimb, on the gastrocnemius muscle, proximal to the lateral saphenous vein).

We completed 60 BM collections (two per animal). Mean weights were 22.0 ± 3.5 g at first and 26.0 ± 2.7 g at second collection point (p-value = 0.164). Anesthesia induction averaged 4.0 ± 0.89 min, aspiration 12.0 ± 3.82 min, and recovery 75.0 ± 24.0 min. No adverse events were noted; three animals discontinued electroacupuncture two minutes early. No postoperative medications beyond standard care were needed, and sutures were removed after seven days without infection or self-injury. Histological evaluation confirmed the presence of myeloid and erythroid precursors and megakaryocytes (Figure 2). Megakaryocyte counts significantly increased in the electroacupuncture group between first and second aspirations (p-value = 0.040) and were significantly higher than controls at the second timepoint (p-value = 0.040). The Sham group showed no significant changes (Figure 2).

**Figure 2:**
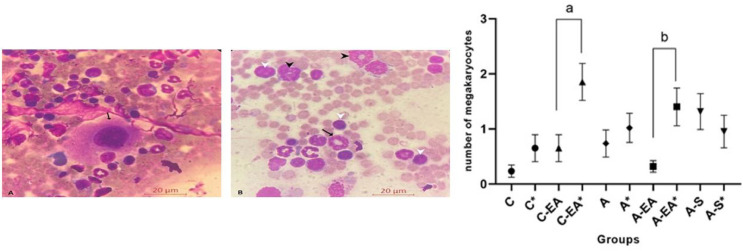
Bone marrow samples from BALB/c mice stained with May-Grünwald-Giemsa. (A) Bone marrow smear showing myeloid and erythroid precursors; a megakaryocyte is indicated by the arrow (× 1000). (B) Bone marrow aspirate highlighting multiple megakaryocytes indicated by arrows (× 100). The graph shows the effect of electroacupuncture on megakaryocyte counts in bone marrow at baseline and after treatment. *Second bone marrow aspiration. A significant increase in megakaryocyte numbers was observed in the electroacupuncture group at the second aspiration. (C) Control group; EA: Electroacupuncture group; SEA: Sham electroacupuncture group.

These initial findings indicate that electroacupuncture stimulates megakaryocyte production in vivo. The experimental design reduced animal use by allowing paired comparisons over time, supporting the 3Rs principles. The iliac crest aspiration method was effective, minimally invasive, and allowed full recovery, making it suitable for longitudinal studies.

Megakaryopoiesis is regulated by thrombopoietin (TPO), a liver-derived cytokine that promotes megakaryocyte differentiation from hematopoietic stem cells and drives platelet production [2]. While we did not measure TPO levels, it is plausible that electroacupuncture may enhance megakaryocyte production by modulating the TPO pathway, which should be evaluated in future studies.

We believe electroacupuncture could emerge as a novel supportive strategy in clinical contexts such as chemotherapy-induced thrombocytopenia or BM failure. Prior studies show that acupuncture influences neuroendocrine and inflammatory pathways [7,8] and promotes stem cell mobilization [9,10], providing mechanistic plausibility to our findings. Importantly, the inclusion of a sham group confirmed that megakaryocyte stimulation was specific to true acupuncture points.

We recognize the preliminary nature of this work and the small sample size. Future investigations should assess TPO modulation, platelet counts, and functional outcomes to elucidate the full hematopoietic impact of electroacupuncture and its translational potential.

In conclusion, our pilot study demonstrates that electroacupuncture stimulates megakaryocyte production in mice and provides a minimally invasive model for repeated BM sampling. We thank the editor for considering this letter and welcome feedback from the Hematology, Transfusion and Cell Therapy readership.

## Conflicts of interest

The authors declare no conflicts of interest.
